# Time to Recovery from Severe Acute Malnutrition and Its Predictors among Admitted Children Aged 6-59 Months at the Therapeutic Feeding Center of Pawi General Hospital, Northwest Ethiopia: A Retrospective Follow-Up Study

**DOI:** 10.1155/2020/8406597

**Published:** 2020-03-11

**Authors:** Amare Wondim, Bethelihem Tigabu, Mengistu Mekonnen Kelkay

**Affiliations:** Department of Pediatric and Child Health Nursing, School of Nursing, College of Medicine and Health Sciences, University of Gondar, Gondar, Ethiopia

## Abstract

**Background:**

Ethiopia is one of the countries in sub-Saharan Africa with the highest rates of severe acute malnutrition. Early recovery is a performance indicator for severe acute malnourished children for the therapeutic feeding. Despite the available interventions to tackle nutritional problems, there is scarce information on time to recovery and its determinants among children with SAM in Ethiopia.

**Objective:**

The study is aimed at assessing time to recovery from severe acute malnutrition and its predictors among admitted children aged 6-59 months at the therapeutic feeding center of Pawi General Hospital, northwest Ethiopia, from January 2013 to December 2017.

**Methods:**

An institution-based retrospective follow-up study was conducted among 398 children aged 6-59 months. The data were collected by using data extraction sheet. The data were cleaned and entered using EpiData version 4.2.0.0 and exported to Stata version 14 statistical software for further analysis. Kaplan-Meier survival curve was used to estimate median nutritional recovery time after initiation of inpatient treatment, and log-rank test was used to compare time to recovery between groups. The Cox proportional regression model was used to identify the predictors of recovery time. Adjusted hazard rate with its 95% CI was reported to show strength of relationship.

**Results:**

The recovery rate was 5.3 per 100 person-day observations, and the median recovery time was 14 days (95% CI: 13–15). The lower chance of early recovery was found among children who were not fully vaccinated (AHR: 0.73 (95% CI: 0.56, 0.96)), while high chance of recovery was found among children who had no anemia (AHR: 1.66 (95% CI: 1.23, 2.23)), TB (AHR: 2.03 (95% CI: 1.11, 3.71)), and malaria infection (AHR: 1.54 (95% CI: 1.09, 2.17)) at admission. *Conclusion and Recommendation*. The overall nutritional recovery rate was below the accepted minimum standard. Children not fully vaccinated and children without malaria, anemia, and TB comorbidities at admission had a higher chance of recovering early from severe acute malnutrition. Hence, treating comorbidities is vital for prompt nutritional recovery.

## 1. Introduction

Severe acute malnutrition (SAM) is defined as very low weight for height < 70%, visible severe wasting, or the presence of nutritional edema. In children aged 6–59 months, mid-upper arm circumference (MUAC) less than 115 mm is also indicative of severe acute malnutrition [1, 2].

Globally, 17 million children are living with severe acute malnutrition; 1 to 2 million die every year; 25 to 30% of the deaths are in many poor countries [2]. Most of the children with SAM live in South Asia and sub-Saharan Africa. Furthermore, it is the most common cause of hospital admissions, accounting for 20% of pediatric hospital admissions in Ethiopia [3].

Ethiopia is still one of the countries with the highest rates of severe acute malnutrition despite a small decline from 12% to 10% over the last 15 years. The prevalence of severe acute malnutrition among under five-year-old children in the country is estimated at 2.9%; in the study where the setting was Benishangul-Gumuz region, it was 3.1% [4]. The annual cost associated with SAM is estimated to be US $262.62 per child in Ethiopia [3]. Besides, delayed discharge from severe acute malnutrition increases the number of children to be treated, medicine cost, and human resource demand, thus imposing economic burden on resource-poor settings [5, 6]. Furthermore, if left untreated, it might result in the impairment of physical, intellectual, social, and adaptive behavior [7, 8].

Studies reported inconsistencies in the rate of recovery from SAM which ranges from 22.1 to 95.36%. In Ethiopia, the rate of recovery is known to be below the accepted international minimum standard of 75%. For instance, Mekele, Bahir Dar, and Gondar reported 22.1% (9), 58.4% [9], and 68.5% [10], respectively. Likewise, studies conducted in Ethiopia on the time to recovery from SAM ranged from 14 to 26 days [9, 11–18]. Shorter recovery time shows an acceptable performance of the treatment and caring process. Recovery time is affected by medical comorbidities like anemia, malaria, dehydration, hypoglycemia, HIV, TB, hypothermia, and lack of child full vaccination. Though these predictors are assumed to delay the time to recovery, some investigators show conflicting results in listing the possible causes of prolonged recovery time.

Although Ethiopia adapted the WHO SAM guideline and has been working for Sustainable Development Goals 2025 under its nutritional strategy goal in order to end hunger, achieve food security, and improve nutrition, only limited information exists regarding the outcome of the SAM treatment provided at the therapeutic feeding center. Ethiopia Demographic and Health Survey (EDHS) reported that the Benishangul-Gumuz region had a high prevalence rate. However, no studies have been conducted in the study area on nutritional recovery. Therefore, this study is aimed at determining the time to recovery from severe acute malnutrition and its predictors among children aged 6-59 months admitted to Pawi General Hospital, northwest Ethiopia.

## 2. Methods and Materials

### 2.1. Study Design, Setting, and Period

**Figure 1:**
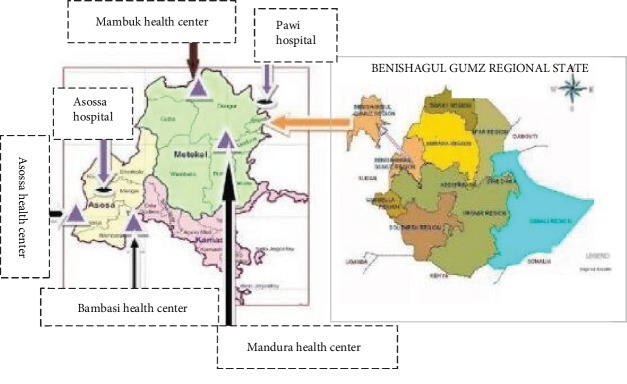
Sketch map of the study setting adapted from a previous study [19].

A hospital-based retrospective follow-up study was conducted on SAM cases enrolled at the hospital from 2013 to 2017. Data was collected from March to April 2018. The study was conducted in the Benishangul-Gumuz regional state, Pawi General Hospital. Pawi is found in the Metekel zone of the Benishangul-Gumuz regional state, 570 km away from Addis Ababa, the capital of Ethiopia. The study site is geographically located at 11.009′N latitude and 36.003′E longitude and at an altitude of 1050 meters above sea level. The mean annual rainfall and maximum temperature of the area are 1555.1 mm and 32°C, respectively. The hospital has different departments that provide specialized services in outpatient, inpatient, and operation theatre departments that serve an estimated 300,000 people in the region, have 200 beds, and have around 1000 inpatients admitted per month on average. The average patient flow to the therapeutic feeding center was 300/year and 25/month for 37 beds (Figure 1).

All children aged 6-59 months and treated for severe acute malnutrition at the therapeutic feeding center of Pawi General Hospital enrolled from January 2013 to December 2017 were included in this study. Children in affected areas were screened for signs of SAM and diagnosed based on anthropometric measurement and examination of the feet for bilateral pitting edema. Children who fulfill the criteria for admission were admitted to the TFC, for example, if the W/H was <70% of the median WHO growth standard, if the MUAC was found to be less than 11 cm, or if the children had bilateral pedal edema. The data were obtained by reviewing records from the inpatient therapeutic feeding registration book and individual follow-up chart.

### 2.2. Sample Size Determination

From a total of 420 children with SAM admitted and treated at the therapeutic feeding center (TFC) of the hospital from January 2013 to December 2017, 398 were selected. After evaluation, 22 of them were excluded as they did not have admission and discharge date. Sample size was determined using Stata version 14; for survival functions, a Cox model based on a retrospective follow-up study conducted in Bahir Dar Felege Hiwot Referral Hospital was used to asses predictors of nutritional recovery time and predictors with SAM having HIV (CHR = 2.35), malaria (CHR = 1.904), and TB (CHR = 2.49) [20] (Table 1).

### 2.3. Data Collection Tool and Procedure

Data were extracted for one month by trained nurses using data extraction sheet prepared in the English language after checking all available data on the severe acute malnutrition inpatient registration book and medical patient charts. The data extraction sheet crosschecked with preestablished known source study variables. Lists of charts of study participants during the study period were taken from the card room, and the charts were coded. Finally, charts which have complete baseline data were selected and variables were recorded.

The data extraction form was developed after reviewing relevant literature from the standard treatment protocol for the management of severe acute malnutrition, SAM registration log book, and monitoring multicharts. It includes child sociodemographic characteristics (age, sex, and residence), anthropometric measurements (height, weight, and MUAC), comorbidities, and treatment outcomes of the child.

### 2.4. Variables of the Study

The dependent variable of the study is time to recovery from SAM (i.e., the event of interest is recovery and the response variable is rate of recovery). The independent variables considered are child sex, age, residence, comorbidities (TB, HIV, cough (pneumonia), anemia, malaria, diarrhea, vomiting, hypoglycemia, dehydration, hypothermia, septic shock, vaccination status, and dermatitis (skin lesion)), type of malnutrition (marasmus, kwashiorkor, and marasmus-kwashiorkor), and routine drugs (folic acid supplement, amoxicillin, and deworming medication).

### 2.5. Operational Definitions

*Recovered*: weight for height greater than or equal to 85% of the median WHO growth chart reference, absence of bilateral pitting edema, and no medical complication [21]

*Time to recovery*: time to recovery was obtained by calculating the difference (in days) from the start of treatment until the child recovered [13]

*Censored*: those who were referred, were nonresponders, were defaulted, and died

*Defaulters*: those who leave the treatment before the child is cured/lost with unknown status, when the patient discontinued before recovery [20]

### 2.6. Data Management and Analysis

Data were entered into EpiData version 4.2.0.0 after checking for completeness, and then the data was cleaned and exported to Stata version 14 for analysis. Descriptive analyses for continuous and categorical data describing the cohort characteristics at baseline and during follow-up were made.

Schoenfeld residual analysis (global test) was used to show the Cox proportional hazard model assumption valid at *P* value = 0.2536. Predictors of time to recovery were identified using bivariable and multivariable Cox proportional hazard models (CPHM). All independent variables that had *P* value less than 0.25 in the bivariable model were considered candidate variables for the multivariable model. The output of the multivariable CPHM is presented using adjusted hazard ratios (AHR) with the respective 95% confidence intervals (CI). Kaplan-Meier survival estimator and log-rank tests were used to estimate median recovery time during the treatment period and to compare time to recovery between groups, respectively. The Cox-Snell plot was used to check the overall model fitness. Multicollinearity was checked using the variance inflation factor.

## 3. Results

### 3.1. Sociodemographic and Baseline Characteristics

Out of 398 children in the cohort, 56.03% were males and 70.35% were from rural areas. The median age was 19 (IQR: 12-32) months. Considering clinical features, 49.75% and 47.24% were marasmus and kwashiorkor, respectively. For marasmic children, the median MUAC was 10 (9.4-10.5) cm and the median length/height and weight of the children were 74 (IQR: 66-81) cm and 6.8 (IQR: 5.5-8.5) kg, respectively (Table 2).

### 3.2. Routine Medication and Treatment

Out of the 398 children, the majority (97.7) received amoxicillin and 85.9% received deworming medication, more than half (57.3%) took vitamin A supplement, and almost all (99.5%) received folic acid supplement. However, only 10.8% of children received IV fluid (Table 3).

### 3.3. Treatment Outcomes, Nutritional Recovery Rate, and Time of Recovery of Children with Severe Acute Malnutrition

**Figure 2:**
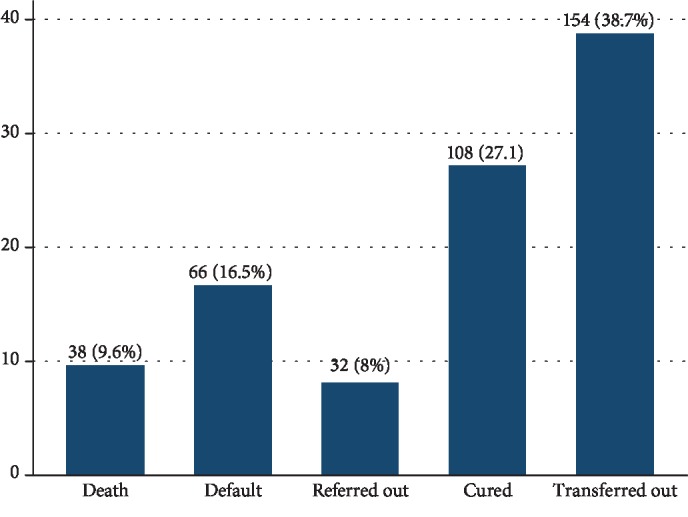
Treatment outcomes of 6–59-month-old children admitted for severe acute malnutrition in the therapeutic feeding center at Pawi General Hospital, northwest Ethiopia, from 2013 to 2017 (*n* = 398).

From 398 children, 262 (65.8%, 95% CI: 60.9-70.5%) recovered, 108 (27.1%) were cured, and 154 (38.7%) transferred out to OTP (Figure 2).

**Figure 3:**
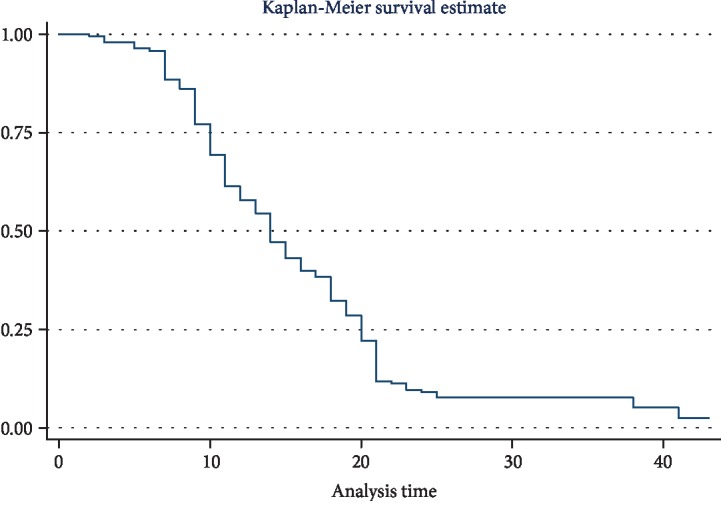
Overall median recovery time of the entire cohort in the therapeutic feeding center at Pawi General Hospital, northwest Ethiopia, from 2013 to 2017.

From those, 65.3% recovered within 28 days with the median time to recovery of 14 day (95% CI: 13–15). Incidence of recovery was 5.3 (95% CI: 4.7–6.0) per 100 person-day observations. Out of these, 33.2% recovered within the second week, followed by 82 (20.6%) within the third week (Figure 3).

However, the recovery rate and time were varied with predictors, including anemia and tuberculosis (Table 4).

### 3.4. Predictors of Time to Recovery from Severe Acute Malnutrition

The multivariable Cox regression analysis showed that malaria, TB, anemia, breast feeding status, and vaccination status were found to be the predictors of time to recovery from SAM. Those who had no malaria at baseline had increased chance of recovery from SAM by 1.54 (AHR: 1.54 (95% CI: 1.09, 2.17)) compared to those who had malaria.

At admission, those who had vaccination status not appropriate for the age had 27% (AHR: 0.73 (95% CI: 0.55, 0.95)) less chance of recovery than their counterparts. Children admitted for SAM with no anemia had 1.66 (AHR: 1.66 (95% CI: 1.23, 2.23)) times more chance of recovery compared to those who had anemia at baseline.

Children without TB infection at admission were 2.03 (AHR: 2.03 (95% CI: 1.11, 3.71)) times more likely to recover from SAM compared to those with TB infection at baseline. Children who had mixed feeding were less likely to recover as compared to those who had exclusive breast feeding (AHR: 0.59 (95% CI: 0.44, 0.81)) (Table 5).

## 4. Discussion

In this study, the recovery rate was 65.8 (95% CI: 60%, 70.5%), which was below the minimum accepted international standard of 75% [22]. It is lower than the finding of studies conducted in various areas of Ethiopia, for example, 77.9% in Debre Markos and Finote Selam [12], 83% in Woliso St. Luke Hospital [13], 87% in Jimma Specialized Referral Hospital [14], and 95.36% in Shebedino [6]. This might be due to differences in human resources and appropriateness of vaccinations for age. However, it is higher than the 22.1% noted in Mekele [11] and 58. 4% noted in Bahir Dar Felege Hiwot Referral Hospital [9].

The median recovery time of 14 days (95% CI: 13, 15) was within the acceptable minimum international standard set for therapeutic feeding centers which is less than 28 days [22]. The finding is in line with those of studies conducted in Woliso and Gedeo zones [13, 23], but it was lower than that in Tigray (21days) [11], Yirgalem (18.6 days) [24], Jimma (21 days) [14], Bahir Dar (16 days) [20], and Karat referral hospitals (26 days) [25]. The discrepancy could be due to differences in the study setting as the latter studies were conducted in referral and specialized hospitals where children with the most severe SAM cases are referred.

In this study, children who had no anemia at admission were more likely to recover early compared to their counterparts. This was congruent with study reports in Bahir Dar Felege Hiwot Referral Hospital and Fasha and Karat [9, 26]. The reason might be that malnourished children are more likely to have micronutrient deficiency particularly iron deficiency anemia and that intervention was not initiated as early as possible as iron damages cell membranes and makes infections worse. Besides, other medical complications also exacerbate time to recovery [27, 28]. Similar to other studies reported elsewhere [9, 24, 29], children without TB infection at admission were 2.03 times more likely to recover from SAM as compared to those with TB infection at baseline. This could be explained by the fact that the presence of malnutrition facilitates the progress of TB infection to an active disease due to its immunosuppressive effects. Moreover, tuberculosis creates a potentially lethal cycle of worsening illness and deteriorating nutritional status, ultimately resulting in a longer recovery time [30]. This finding revealed that severely malnourished children with no malaria infection recovered early compared to children with malaria infection. This is supported by studies conducted in Niger, Amazon, and Rwanda [25, 31, 32]. The reason might be that malaria impairs gluconeogenesis and increases energy consumption, induces malabsorption, predisposes one to loss of immunity, and results in inability respond to standard treatments [32, 33].

Severely malnourished children who had vaccination status not appropriate for the age were 27% less likely to recover earlier than their counterparts. The finding is supported by results in developing countries [34]. The impact of severe acute malnutrition will be worsened as innate and adaptive immune dysfunction leads to immune dysregulation [34, 35].

Children who had history of mixed feeding were 41% less likely to recover earlier than children who had history of exclusive breast feeding. The finding is in line with a study reported by World Health Organization previously [36] and a finding from Dire Dawa, eastern Ethiopia [37]. Mixed feeding may impose additional pathogen and complicate the management of children with SAM.

## 5. Strengths and Limitations

The strength of the study was the use of the advanced statistical analysis model. Further, this study is one among few studies in Ethiopia particularly novice for the study setting that were conducted to assess treatment outcomes and factors affecting time to recovery among 6–59-month-old children with SAM admitted at the Pawi General Hospital stabilization center. Due to the nature of the retrospective and secondary data, incomplete records were seen in some variables. We were unable to explore other sociodemographic characteristics, such as maternal and paternal educational status.

## 6. Conclusion

In the study area, the overall recovery rate was low. Children whose vaccination status was not appropriate for age and who had malaria, anemia, TB comorbidities, and history of mixed feeding at admission were predictors associated with time to recovery from severe acute malnutrition. Thus, emphasis should be given to prompt and timely management of malaria, anemia, and TB and more importantly to universal vaccination coverage appropriate for age.

## Figures and Tables

**Table 1:** 

Predictors	Power	Recovery	Withdrawal	CHR	Sample size
HIV	90%	0.519	0.481	2.35	215
TB	90%	0.519	0.481	2.49	188
Malaria	90%	0.519	0.481	1.904	379

**Table 2:** Sociodemographic and baseline characteristics of 6–59-month-old children with severe acute malnutrition in the therapeutic feeding center at Pawi General Hospital, northwest Ethiopia (2013-2017) (*n* = 398).

Variable	Character	Frequency (%)
Sex	Male	223 (56.0)
	Female	175 (44.0)

Residence	Rural	281 (70.6)
	Urban	117 (29.4)

Age of the child (months)	6-11	97 (24.4)
	12-23	116 (29.2)
	24-35	96 (24.0)
	36-47	42 (10.6)
	48-59	47 (11.8)

SAM diagnosis type	Marasmus	198 (49.8)
	Kwashiorkor	188 (47.2)
	Marasmus-kwashiorkor	12 (3.0)

Vaccination status	Appropriate for age	166 (41.7)
	Not appropriate for age	147 (36.9)
	Not documented	15 (3.8)
	Not vaccinated	70 (17.6)

Hypoglycemia	Absent	78 (95.0)
	Present	20 (5.0)

Dermatitis	Absent	348 (87.4)
	Present	50 (12.6)

Dehydration	No	190 (47.7)
	Yes	208 (52.3)

Vomiting	Absent	261 (65.6)
	Present	137 (34.4)

Diarrhea	Absent	191 (48.0)
	Present	207 (52.0)

HIV	Yes	1 (0.3)
	No	397 (99.7)

Septic shock	Yes	18 (4.5)
	No	380 (95.5)

Malaria	Yes	76 (19.15)
	No	322 (80.9)

Anemia	Yes	137 (34.4)
	No	261 (65.6)

Pneumonia	Yes	195 (49. 0)
	No	203 (51.0)

TB	Yes	27 (6.8)
	No	371 (93.2)

Hypothermia	Yes	11 (2.8)
	No	387 (97.2)

**Table 3:** Routine medication and supplementation for 6–59-month-old children with severe acute malnutrition in the therapeutic feeding center of Pawi General Hospital, northwest Ethiopia (2013-2017) (*n* = 398).

Characteristics	Frequency (%)
Amoxicillin	
Yes	389 (97.7)
No	9 (2.3)
Folic acid	
Yes	396 (99.5)
No	2 (0.5%)
Deworming	
Yes	342 (85.9)
No	56 (14.1)
Vitamin A	
Yes	228 (57.3)
No	170 (42.7)
NG tube feeding	
Yes	123 (30.9)
No	275 (69.1)
IV fluid	
Yes	43 (10.8)
No	355 (89.2)
Breast feeding status	
Exclusive breast feeding	277 (69.6)
Mixed	121 (30.4)
Blood transfusion	
Yes	13 (3.3)
No	385 (96.7)
Antimalarial drug	
Yes	37 (9.3)
No	361 (90.7)

**Table 4:** Recovery time and rate from SAM by predictors of among children with SAM managed at Pawi General Hospital from 2013 to 2017.

Variable	Characteristics	Subjects	Recovery rate (per 100 person-days)	Median (IQR) in days	95% CI
Anemia	Yes	137	4.2	19 (11, 21)	19 (16, 20)
	No	261	6.0	12 (9, 18)	12 (11, 14)
	Total	398	5.3	14 (10, 20)	14 (13, 15)

Vaccination status	Appropriate	166	6.5	13 (9, 19)	13 (11, 14)
	Not appropriate	232	4.4	15 (10, 21)	15 (14, 18)
	Total	398	5.3	14 (10, 20)	14 (13, 15)

TB	Yes	27	3.5	21 (12, 23)	21 (12, 23)
	No	371	5.5	14 (10, 20)	14 (13, 15)
	Total	398	5.3	14 (10, 20)	14 (13, 15)

Malaria	Yes	76	4.1	19 (14, 21)	19 (18, 21)
	No	322	5.7	13 (9, 19)	13 (11, 14)
	Total	398	5.3	14 (10, 20)	14 (13, 15)

**Table 5:** Predictors of recovery time from severe acute malnutrition among 6–59-month-old children admitted in the therapeutic feeding center of Pawi General Hospital, northwest Ethiopia (2013-2017).

Characteristics	Recovered	Censored	CHR (95% CI)	*P* value	AHR (95% CI)
Residence					
Rural	174	106	1		1
Urban	88	30	1.35 (1.05, 1.75)	0.0139	1.23 (0.93, 1.61)
Age					
6-23	131	82	1		1
24-59	131	54	1.19 (0.80, 1.30)	0.200	1.2 (0.92, 1.57)
Malaria					
Yes	46	29	1		1
No	216	107	1.90 (1.38, 2.62)	**0.001**	**1.54 (1.09, 2.17)**
Diarrhea					
Absent	128	63	1		1
Present	134	73	0.81 (0.64, 1.04)	0.072	1.17 (0.88, 1.55)
TB					
Yes	13	14	1		1
No	249	122	2.05 (1.17, 3.60)	**0.0053**	**2.03 (1.11, 3.71)**
Pneumonia					
Absent	147	55	1		1
Present	115	82	0.76 (0.59, 0.97)	0.018	1.02 (0.77, 1.34)
Dermatitis					
Absent	222	125	1		1
Present	40	11	1.77 (1.25, 2.49)	0.139	1.36 (0.93, 2.00)
Hypoglycemia					
Absent	257	120	1		1
Present	5	16	0.39 (0.14, 1.05)	0.144	0.41 (0.15, 1.12)
Vaccination status					
Appropriate for age	138	28	1		1
Not appropriate for age	108	124	0.68 (0.53, 0.87)	0.0009	**0.73 (0.55, 0.95)**
Anemia					
Yes	79	58	1		1
No	183	78	1.88 (1.44, 2.47)	**0.001**	**1.66 (1.23, 2.23)**
Vomiting					
Yes	177	84	0.73 (0.56, 0.95)	**0.122**	0.85 (0.64, 1.13)
No	85	52	1		1
Breast feeding status					
Exclusive	200	77	1		**1**
Mixed	62	59	0.55 (0.41, 0.73)	**0.001**	**0.59 (0.44, 0.81)**
ReSoMal					
Yes	163	74	1		1
No	99	62	1.38 (1.07, 1.78)	**0.114**	1.27 (0.94, 1.69)

The global proportionality assumption test (*P* value = 0.2536).

## Data Availability

The datasets used and/or analyzed and in which conclusions are drawn during the current study are included in the manuscript.

## Abbreviations

AHR: Adjusted hazard ratios
CPHM: Cox proportional hazard models
CHR: Crude hazard ratio
CI: Confidence intervals
GDP: Gross domestic product
EDHS: Ethiopia Demographic and Health Survey
HIV: Human immunodeficiency virus
IQR: Interquartile range
IV: Intravenous
MUAC: Mid-upper arm circumference
NG: Nasogastric tube
SAM: Severe acute malnutrition
SD: Standard deviation
TB: Tuberculosis
TFC: Therapeutic feeding center
WHO: World Health Organization
*χ*^2^: Chi-square.
